# Gestational Age and Birth Outcomes in Term Singleton Pregnancies Conceived With Infertility Treatment

**DOI:** 10.1001/jamanetworkopen.2023.28335

**Published:** 2023-08-11

**Authors:** Ira Hamilton, Nicole Martin, James Liu, Emily DeFranco, Robert Rossi

**Affiliations:** 1 Division of Maternal-Fetal Medicine, Department of Obstetrics and Gynecology, University of Cincinnati College of Medicine, Cincinnati, Ohio

## Abstract

**Importance:**

Despite the increased perinatal risks associated with pregnancies conceived with infertility treatment, there are no recommendations for timing of delivery among this at-risk population.

**Objective:**

To identify the gestational age at which the ongoing risks of stillbirth are optimally balanced with the risks of neonatal comorbidities and infant deaths in term singleton pregnancies conceived with infertility treatment.

**Design, Setting, and Participants:**

This cohort study used birth and death data from January 1, 2014, to December 31, 2018, in the US obtained from the National Center for Health Statistics. Singleton pregnancies conceived with infertility treatment delivered at term (37-42 weeks’ gestation) were eligible for inclusion. The exclusion criteria were deliveries at less than 37 weeks’ or at least 43 weeks’ gestation and pregnancies with unknown history of infertility treatment, congenital anomalies, pregestational diabetes, pregestational hypertension, gestational hypertension, and preeclampsia. Data were analyzed from July 22, 2022, to June 24, 2023.

**Exposure:**

Gestational age at delivery between 37 and 42 weeks.

**Main Outcomes and Measures:**

The primary outcome was optimal timing of delivery. To ascertain this timing, the risk of delivery (rate of neonatal morbidity and infant death) at a given gestational week was compared with the risk of delivery in the subsequent week of gestation for an additional week (rate of stillbirth during the given week per 10 000 ongoing pregnancies plus rate of neonatal morbidity and infant death in the subsequent week of gestation per 10 000 deliveries). The rates of stillbirth, neonatal morbidity, and infant death (within 1 year of life) were compared at each week. Neonatal morbidity included an Apgar score of 3 or lower at 5 minutes, requirement of ventilation for 6 hours or more, neonatal intensive care unit admission, and seizures.

**Results:**

Of the 178 448 singleton term pregnancies conceived with infertility treatment (maternal mean [SD] age, 34.2 [5.2] years; mean [SD] gestational age, 39.2 [1.2] weeks; 130 786 [73.5%] were non-Hispanic White patients). The risk of delivery in the subsequent week of gestation was lower than the risk of delivery at both 37 weeks (628 [95% CI, 601-656] vs 1005 [95% CI, 961-1050] per 10 000 live births) and 38 weeks (483 [95% CI, 467-500 vs 625 [95% CI, 598-652] per 10 000 live births). The risks of delivery in subsequent week of gestation significantly exceeded the risk of delivery at 39 weeks (599 [95% CI, 576-622] vs 479 [95% CI, 463-495] per 10 000 live births) and were not significant at 40 weeks (639 [95% CI, 605-675] vs 594 [95% CI, 572-617] per 10 000 live births) and 41 weeks (701 [95% CI, 628-781] vs 633 [95% CI, 599-669] per 10 000 live births).

**Conclusions and Relevance:**

Results of this study suggest that, in pregnancies conceived with infertility treatment, delivery at 39 weeks provided the lowest perinatal risk when comparing risk of delivery at this week of gestation vs the subsequent week of gestation.

## Key Points

**Question:**

What gestational age at delivery is associated with the best perinatal outcomes in term singleton pregnancies conceived with infertility treatment?

**Findings:**

In this cohort study of 178 448 singleton term pregnancies conceived with infertility treatment, at 39 weeks’ gestation the risk of delivery (neonatal morbidity and infant death) during a given week of gestation was significantly lower than the risk of delivery (stillbirth and neonatal morbidity and infant death) in the subsequent week of gestation.

**Meaning:**

Findings from this study suggest that in term singleton pregnancies conceived with infertility treatment, there is an increased risk of adverse perinatal outcomes with both early-term and late-term delivery.

## Teaser

This cohort study evaluates the rates of stillbirth, neonatal morbidity, and infant death associated with delivery timing in pregnancies aided by infertility treatment.

## Introduction

According to the National Center for Health Statistics (NCHS), between 2015 and 2019, 12.2% of females aged 15 to 49 years received infertility therapy.^1^ Infants conceived via assisted reproductive technology (ART), specifically, accounted for approximately 2% of births in the US in 2018.^2^ Compared with spontaneous pregnancies (not requiring infertility treatment), pregnancies conceived with ART have been associated with increased risk of low birth weight, perinatal mortality, and stillbirth,^3^ It is unclear, however, whether these associations are due to the mode of conception or the underlying conditions necessitating ART.^4^

Despite these risks, neither the Society for Maternal-Fetal Medicine nor the American College of Obstetricians and Gynecologists provides a recommendation for the timing of delivery in pregnancies conceived with infertility treatment; instead, these organizations recommend shared and individualized decision-making given the absence of data.^5,6^ Ideal time of delivery optimizes outcomes by balancing the risks of stillbirth of a continuing pregnancy against poor perinatal outcomes, such as low Apgar scores, neonatal intensive care unit (NICU) admission, respiratory distress, seizures, and infant death, associated with early-term delivery. In this study, we aimed to identify the gestational age at which the ongoing risks of stillbirth are optimally balanced with the risks of neonatal comorbidities and infant deaths in term singleton pregnancies conceived with infertility treatment.

## Methods

We conducted a population-based retrospective cohort study in a contemporary US population of term pregnancies conceived with infertility treatment or ART. Deidentified, publicly available data were obtained from the NCHS National Vital Statistics System. The data were abstracted from the period or cohort’s Linked Birth/Infant Death and Fetal Death Records from January 1, 2014, to December 31, 2018. The NCHS reviews, links, and edits the mortality and natality data provided by the states, which form the basis of official US birth and death statistics after the data are coded uniformly and undergo quality control standards.^7^ The University of Cincinnati Institutional Review Board deemed this study exempt from review and informed consent requirement because it did not meet the criteria for human participant research. We followed the Strengthening the Reporting of Observational Studies in Epidemiology (STROBE) reporting guideline.^8^

For the primary objective of this study, we determined the balance of the risks of delivery in a given week of gestation with risks of delivery in the subsequent week of gestation at term. The risk of delivery was defined as the composite rate of infant death and neonatal morbidity incurred at a given week of gestation per 10 000 deliveries. The risk of delivery in the subsequent week of gestation was defined as the combined rate of stillbirth at a given week of gestation per 10 000 ongoing pregnancies plus the rate of infant death and neonatal morbidity in the subsequent week of gestation per 10 000 deliveries. This definition has previously been described, but with the addition of neonatal morbidity in the risk assessment to more fully capture the risk of delaying delivery.^9^

The broad categorization of infertility treatment in the NCHS databases included 2 separate subcategories: use of fertility enhancing drugs, artificial insemination, or intrauterine insemination and use of ART (eg, in vitro fertilization [IVF], gamete intrafallopian transfer, or zygote intrafallopian transfer). These definitions reflect those used by the American Society for Reproductive Medicine.^10,11^ Both subcategories could be reported in the same birth. Thus, analyses were performed for pregnancies conceived with any infertility treatment and a separate subgroup analysis was performed for those conceived specifically with ART.

Exclusion criteria included deliveries at less than 37 weeks’ or at least 43 weeks’ gestation and pregnancies with multiple gestations, preexisting or gestational diabetes, gestational or chronic hypertension, preeclampsia or eclampsia, and unknown history of diabetes or chronic hypertension. Pregnancies that were complicated by a fetus with congenital anomalies were excluded if the anomaly was reported in the natality file or was reported as the cause of death in the mortality or fetal death data files, according to the *International Statistical Classification of Diseases, Tenth Revision,* codes.

Gestational age was defined using the standard clinical practice for gestational age estimation: the best obstetric estimate in the birth record, which is calculated using the last menstrual period and other clinical and ultrasonography parameters. At each week of gestation, the rate of stillbirth was calculated as the number of antepartum or intrapartum stillbirths at that gestation per 10 000 ongoing pregnancies. The rate of infant death was calculated as the number of infants born at a given week of gestation who died within 1 year of life, per 10 000 live births, at that same gestation. Infant death was chosen because it shares similar risk factors with stillbirth, varies with gestational age at term, and contains the complications of perinatal risk more fully in the setting of available advanced neonatal intensive care.^12^ Small for gestational age was defined as weight of less than the 10th percentile for gestational age.^13^ We used prepregnancy height and weight to determine maternal obesity, which was a body mass index (calculated as weight in kilograms divided by height in meters squared) of 30 or higher.^14^ Self-reported race and ethnicity in the patient medical record were used to generate these categories: Hispanic, non-Hispanic Black, non-Hispanic White, and other (including American Indian or Alaskan, Asian, and Pacific Islander). Race and ethnicity data were collected and analyzed to accurately describe the study population and to evaluate possible racial and ethnic disparities.

An Apgar score of 3 or lower at 5 minutes, ventilation of 6 hours or more, seizures, or NICU admission were considered to be a neonatal morbidity. Each of these adverse neonatal outcomes has been associated with serious morbidity and long-term disability.^12,15,16^ The infant death rate was defined as the number of infant deaths per 10 000 live births at each week of gestation. With care given to ensure that cases were not double-counted, the rate of neonatal morbidity or infant death was calculated as the number of morbidity or mortality events at a given gestation per 10 000 live births.

In this study using a modified, previously studied risk-assessment method, we examined morbidity and mortality at each week of term gestation to identify the optimal time of delivery (the primary outcome) in pregnancies conceived with infertility treatment or specifically ART.^9^ The risk of delivery at each week (ie, the rate of neonatal morbidity or infant death) was compared with the risk of delivery in the subsequent week (ie, the rate of stillbirth during that week plus the rate of neonatal morbidity or infant death in the subsequent week).

### Statistical Analysis

We performed statistical calculations with Stata 15 (StataCorp LLC), including proportions, relative risks (RRs), and 95% CIs. The χ^2^ test was used to compare proportions of independent variables, and analysis of variance was used to compare means, including neonatal birth weight, maternal body mass index, and age. Statistical significance was defined as reaching a nominal *P* < .05 or if the 95% CI did not cross 1. Data were analyzed from July 22, 2022, to June 24, 2023.

## Results

The 340 728 pregnancies conceived with infertility treatments (maternal mean [SD] age, 34.2 [5.2] years; mean [SD] gestational age, 39.2 [1.2] weeks) between 2014 and 2018 corresponded with a 1.7% prevalence rate and was consistent with the increase of infertility treatments in the US. After exclusion of deliveries at less than 37 weeks’ or at least 43 weeks’ gestation (n = 99 338) and pregnancies with multifetal gestations (n = 37 457); pregestational diabetes, hypertension, or unknown history of either (n = 8295); pregnancy-induced hypertension (n = 16 429); or fetal congenital anomalies (n = 761) (eFigure 1 in Supplement 1), 178 448 singleton term pregnancies were included in the study. The rate of stillbirth was 0.14% (n = 248), and the rate of infant death was 0.07% (n = 128).

Applying the exclusion criteria, we found that pregnancies conceived with infertility treatment compared with spontaneous pregnancies had different characteristics, including higher rates of non-Hispanic White patients, higher proportion of patients being 35 years or older, and higher rates of nulliparity (eTable 1 in Supplement 1). Moreover, spontaneous pregnancies had lower rates of preterm delivery (PTD) compared with pregnancies conceived with infertility treatment (PTD at <37 weeks’ gestation: 9.1% vs 11.0%; PTD at <34 weeks’ gestation: 2.6% vs 3.5%). Table 1 describes the maternal characteristics among pregnancies conceived through infertility treatment by the outcomes of stillbirth, infant death, or live birth. Neonatal birth weight was lower overall, and the small for gestational age rate was higher in the stillbirth and infant death cohorts vs the live birth cohort (28.2% and 23.4% vs 8.3%). The rates of stillbirth and infant death were 11.7% and 10.9%, respectively, among Hispanic patients; 6.1% and 5.5%, respectively, among non-Hispanic Black patients; and 68.2% and 67.2%, respectively, among non-Hispanic White patients.

**Table 1.** Baseline Patient Characteristics in Pregnancies Conceived With Infertility Treatment

Characteristic	Outcome of pregnancies, No. (%)		
	Stillbirth (n = 248)	Infant death (n = 128)	Live birth (n = 178 072)
Maternal race and ethnicity^a^			
Hispanic	29 (11.7)	14 (10.9)	13 914 (7.8)
Non-Hispanic Black	15 (6.1)	7 (5.5)	7069 (4.0)
Non-Hispanic White	169 (68.2)	86 (67.2)	130 786 (73.5)
Other^b^	26 (10.5)	18 (14.1)	22 766 (12.8)
Maternal age, mean (SD), y	33.9 (4.9)	34.0 (5.8)	34.2 (5.2)
Maternal age group, y			
<18	0 (0)	1 (0.8)	31 (0)
18-34	146 (58.9)	69 (53.9)	96 418 (54.2)
≥35	102 (41.1)	58 (45.3)	81 623 (45.8)
Nulliparity	148 (59.7)	73 (57.0)	107 668 (60.5)
<High school diploma	5 (2.2)	2 (1.6)	2235 (1.3)
Married	NA	109 (85.2)	157 083 (88.2)
Women, Infants and Children program enrollment	11 (5.1)	7 (5.5)	10 377 (5.9)
Prenatal care			
No prenatal care	1 (0.4)	1 (0.8)	253 (0.1)
Late initiation: >20 wks’ gestation	10 (4.3)	4 (3.2)	6929 (4.0)
Tobacco use	3 (1.3)	2 (1.6)	1390 (0.8)
BMI, mean (SD)	27.9 (6.7)	26.6 (6.9)	25.9 (5.9)
Obesity: BMI ≥30	72 (30.8)	29 (23.8)	35 885 (20.5)
Gestational age, mean (SD), wk	38.7 (1.4)	39.1 (1.3)	39.2 (1.2)
Birth weight, mean (SD), g	3009 (703)	3213 (653)	3416 (467)
SGA	70 (28.2)	30 (23.4)	14 684 (8.3)

Abbreviations: BMI, body mass index (calculated as weight in kilograms divided by height in meters squared); NA, not applicable (not recorded in the fetal death data); SGA, small for gestational age.

^a^ Race and ethnicity data were self-reported by patients and obtained from the patient medical record.

^b^ Other includes American Indian or Alaskan, Asian, and Pacific Islander.

At each week of term gestation (37-42 weeks), the observed number of ongoing pregnancies and live births along with rates of stillbirths, infant deaths or neonatal morbidities, and composite rates of infant deaths or neonatal morbidities with delivery in the subsequent week of gestation are provided for pregnancies conceived with infertility treatment (Table 2) or ART (Table 3). For pregnancies conceived with infertility treatment, the rate of stillbirth per 10 000 ongoing pregnancies increased with each week of term gestation, being lowest at 37 weeks (3.1; 95% CI, 2.3-4.0) and highest at 42 weeks (21.0; 95% CI, 11.0-39.7). For pregnancies conceived specifically with ART, a similar pattern was seen: 2.5 (95% CI, 1.7-3.7) per 10 000 ongoing pregnancies at 37 weeks vs 23.6 (95% CI, 10.8-51.4) per 10 000 ongoing pregnancies at 42 weeks. The rate of infant death per 10 000 live births followed a U-shaped pattern, being lowest at 39 weeks in both pregnancies conceived with infertility treatment (6.2; 95% CI, 4.2-9.1) and with ART (5.2; 95% CI, 3.2-8.1). The absolute risk differences in composite morbidity or mortality between delivery in the subsequent week of gestation and delivery at 39 weeks were 120 per 10 000 live births for the infertility treatment cohort and 154 per 10 000 live births for the ART cohort.

**Table 2.** Stillbirths, Infant Deaths, and Neonatal Morbidities by Gestational Age in Pregnancies Conceived With Infertility Treatment^a^

Wk of term gestation	Ongoing pregnancies, No.	Stillbirths per 10 000 ongoing pregnancies (95% CI)	Live births, No.	Infant deaths per 10 000 live births (95% CI)	Composite rate of infant deaths with delivery in subsequent wk of gestation^b^	Neonatal morbidities per 10 000 live births (95% CI)^c^	Infant deaths or neonatal morbidities per 10 000 live births (95% CI)^c^	Composite rate of infant deaths or neonatal morbidities with delivery in subsequent wk of gestation^c^^,^^d^
37	178 448	3.1 (2.3-4.0)	17 348	10.4 (6.6-16.4)	9.4 (6.5-13.5)	1002 (958-1047)	1005 (961-1050)	628 (601-656)
38	161 045	4.2 (3.2-5.2)	30 225	6.3 (4.0-9.8)	10.8 (8.6-13.6)	621 (594-649)	625 (598-652)	483 (467-500)
39	130 753	4.7 (3.6-5.9)	65 960	6.7 (5.0-9.0)	10.8 (8.1-14.5)	476 (460-492)	479 (463-495)	599 (576-622)
40	64 732	5.9 (4.2-8)	41 849	6.2 (4.2-9.1)	15.6 (10.8-22.4)	591 (569-614)	594 (572-617)	639 (605-675)
41	22 845	7.9 (4.9-12.4)	18 532	9.7 (6.2-15.4)	14.9 (7.0-14.9)	628 (594-664)	633 (599-669)	701 (628-781)
42	4295	21.0 (11.0-39.7)	4286	7.0 (2.4-20.6)	NA	693 (621-773)	693 (621-773)	NA

Abbreviation: NA, not applicable.

^a^ Infertility treatment included the use of fertility-enhancing drugs, artificial insemination, intrauterine insemination, or assisted reproductive technology (in vitro fertilization, gamete intrafallopian transfer, or zygote intrafallopian transfer).

^b^ Composite rate of infant death with delivery in subsequent week of gestation was defined as the rate of stillbirth at this gestational age per 10 000 ongoing pregnancies plus the rate of infant death during the subsequent week of gestation per 10 000 deliveries (95% CI).

^c^ Neonatal morbidity was defined as the composite of neonatal intensive care unit admission, ventilatory support for more than 6 hours, seizures, or an Apgar score of 3 or lower at 5 minutes.

^d^ Composite rate of infant death or neonatal morbidity with delivery in subsequent week of gestation was defined as the rate of stillbirth at this gestational age per 10 000 ongoing pregnancies plus rate of infant death or neonatal morbidity during the subsequent week of gestation per 10 000 deliveries (95% CI).

**Table 3.** Stillbirths, Infant Deaths, Neonatal Morbidities, and Expectant Management by Gestational Age in Pregnancies Conceived With Assisted Reproductive Technology^a^

Wk of term gestation	Ongoing pregnancies, No.	Stillbirths per 10 000 ongoing pregnancies (95% CI)	Live births, No.	Infant deaths per 10 000 live births (95% CI)	Composite rate of infant deaths with delivery in subsequent wk of gestation^b^	Neonatal morbidities per 10 000 live births (95% CI)^c^	Infant deaths or neonatal morbidities per 10 000 live births (95% CI)^c^	Composite rate of infant deaths or neonatal morbidities with delivery in subsequent wk of gestation^c^^,^^d^
37	94 776	2.5 (1.7-3.7)	9631	12.5 (7.1-21.7)	8.8 (5.2-14.7)	1064 (1004-1127)	1068 (1008-1132)	668 (631-708)
38	85 145	4.7 (3.4-6.3)	16 024	6.2 (3.3-11.4)	9.9 (7.1-13.8)	663 (625-702)	666 (628-706)	515 (492-539)
39	69 121	5.2 (3.7-7.2)	34 712	5.2 (3.2-8.1)	11.6 (7.9-17.1)	508 (486-532)	510 (488-534)	664 (631-697)
40	34 409	5.2 (3.3-8.2)	21 815	6.4 (3.8-10.7)	16.2 (10.0-26.2)	655 (623-689)	658 (626-692)	663 (616-713)
41	12 594	7.9 (4.3-14.6)	10 055	10.9 (6.1-19.5)	15.9 (6.2-40.5)	653 (607-703)	657 (611-708)	693 (601-799)
42	2539	23.6 (10.8-51.4)	2539	7.9 (2.1-28.6)	NA	685 (593-790)	685 (593-790)	NA

Abbreviation: NA, not applicable.

^a^ Assisted reproductive technology included the use of in vitro fertilization, gamete intrafallopian transfer, and zygote intrafallopian transfer.

^b^ Composite rate of infant deaths with delivery in subsequent week of gestation was defined as the rate of stillbirth at this gestational age per 10 000 ongoing pregnancies plus rate of infant death during the subsequent week of gestation per 10 000 deliveries (95% CI).

^c^ Neonatal morbidity was defined as the composite of neonatal intensive care unit admission, ventilatory support for more than 6 hours, seizures, or an Apgar score of 3 or lower at 5 minutes.

^d^ Composite rate of infant deaths or neonatal morbidities with delivery in subsequent week of gestation was defined as the rate of stillbirth at this gestational age per 10 000 ongoing pregnancies plus the rate of infant death or neonatal morbidity during the subsequent week of gestation per 10 000 deliveries (95% CI).

**Figure 1.**
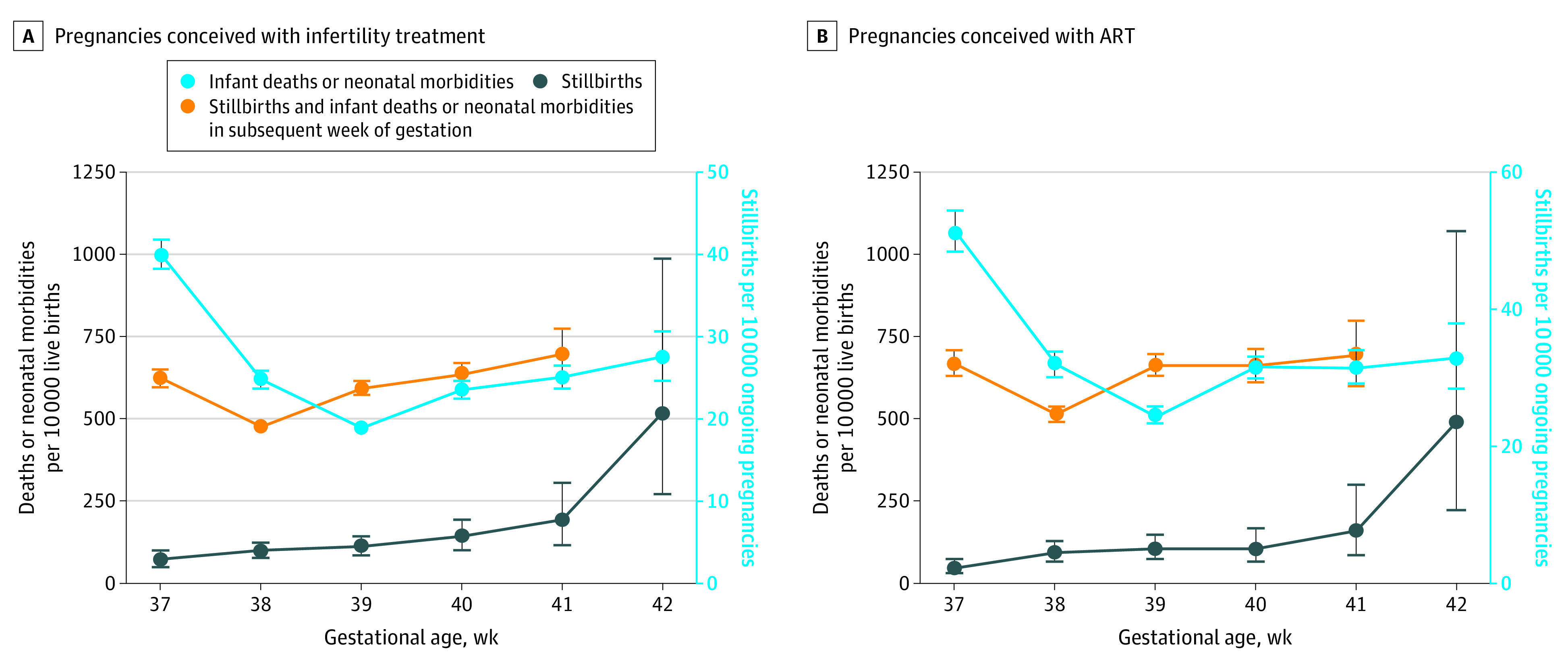
Comparison of Rate of Infant Deaths or Neonatal Morbidities vs Rate of Stillbirths and Infant Deaths or Neonatal Morbidities in the Subsequent Week of Gestation in Term Pregnancies Conceived With Infertility Treatment or Assisted Reproductive Technology (ART) Error bars represent 95% CIs.

Figure 1 compares the risk of delivery in a given week of gestation with the risk of delivery in the subsequent week of gestation in pregnancies conceived with infertility treatment (Figure 1A) and ART (Figure 1B). The risk of delivery in the subsequent week of gestation was the combined rate of stillbirth at a given week of gestation per 10 000 ongoing pregnancies and the rate of neonatal morbidity and infant death incurred from delivery in the subsequent week of gestation per 10 000 live births. In pregnancies conceived with infertility treatment, the risk of delivery in the subsequent week of gestation was lower than the risk of delivery at both 37 weeks (628 [95% CI, 601-656] vs 1005 [95% CI, 961-1050] per 10 000 live births) and 38 weeks (483 [95% CI, 467-500 vs 625 [95% CI, 598-652] per 10 000 live births). The risks of delivery in the subsequent week of gestation significantly exceeded the risk of delivery at 39 weeks (599 [95% CI, 576-622] vs 479 [95% CI, 463-495] per 10 000 live births) but were not significant at 40 weeks (639 [95% CI, 605-675] vs 594 [95% CI, 572-617] per 10 000 live births) and 41 weeks (701 [95% CI, 628-781] vs 633 [95% CI, 599-669] per 10 000 live births). In pregnancies conceived with ART, the risk of delivery in the subsequent week of gestation was lower than the risk of delivery at both 37 weeks (668 [95% CI, 631-708] vs 1068 [95% CI, 1008-1132] per 10 000 live births) and 38 weeks (515 [95% CI, 492-539] vs 666 [95% CI, 628-706] per 10 000 live births). The risks of delivery in the subsequent week of gestation significantly exceeded the risk of delivery at 39 weeks (664 [95% CI, 631-697] vs 510 [95% CI, 488-534] per 10 000 live births) and were not significant at 40 weeks (663 [95% CI, 616-713] vs 658 [95% CI, 626-692] per 10 000 live births) and 41 weeks (693 [95% CI, 601-799] vs 657 [95% CI, 611-708] per 10 000 live births).

**Figure 2.**
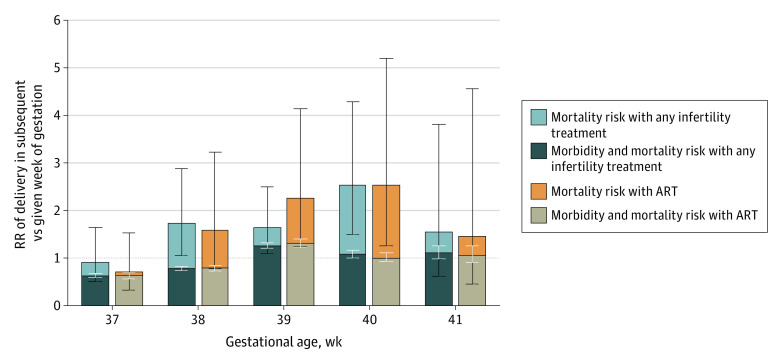
Comparison of Relative Risk (RR) of Delivery in Subsequent Week of Gestation vs Each Week of Gestation in Pregnancies Conceived With Any Infertility Treatment or Assisted Reproductive Technology (ART) White error bars represent 95% CIs for the morbidity and mortality risks, and black error bars represent 95% CIs for the mortality risks.

The overall relative composite mortality and morbidity risk of delivery in the subsequent week of gestation compared with a given week of gestation increased with advancing gestation (Figure 2). In pregnancies conceived with infertility treatment, the comparative RR of delivery in the subsequent vs given week of gestation after accounting for both morbidity and mortality was lowest at 37 weeks’ (RR, 0.62; 95% CI, 0.59-0.66) and 38 weeks’ (RR, 0.77; 95% CI, 0.73-0.82) gestation (eTable 2 in Supplement 1). At 39 weeks’ gestation and later, the comparative RR of delivery in the subsequent vs given week of gestation exceeded the risk of delivery (RR, 1.25; 95% CI, 1.19-1.31). In these same pregnancies, the comparative mortality risk of delivery in the subsequent vs given week of gestation was higher at 38 weeks’ gestation and later (38 weeks: RR, 1.72 [95% CI, 1.04-2.86]; 39 weeks: RR, 1.63 [95% CI, 1.08-2.47]; 40 weeks: RR, 2.51 [95% CI, 1.48-4.26]).

Similarly, among pregnancies conceived with ART, the relative composite morbidity and mortality risk of delivery in the subsequent week of gestation also exceeded the risk of delivery at 39 weeks’ gestation (RR, 1.30; 95% CI, 1.22-1.39) (eTable 3 in Supplement 1). The comparative mortality risk of delivery in the subsequent week of gestation was not significantly higher than that at 38 weeks’ gestation (RR, 1.58; 95% CI, 0.78-3.20) and was significantly higher after 39 weeks’ (RR, 2.24; 95% CI, 1.23-4.10) and 40 weeks’ gestation (RR, 2.52; 95% CI, 1.24-5.15).

Thematically categorized causes of infant death and initiating causes of stillbirth among pregnancies conceived with infertility treatment, stratified by gestational age of delivery, are provided in eTables 4 and 5 in Supplement 1, respectively. eFigure 2 in Supplement 1 shows the relative contributions of these causes of stillbirths and infant deaths among term gestations (37-42 weeks).

## Discussion

Guidance for delivery timing is immensely important to optimally balance the risk of stillbirth with the risks of infant death and neonatal morbidity due to early-term or late-term delivery. This cohort study provides an evidenced-based recommendation of delivery at 39 weeks’ gestation for pregnancies conceived with infertility treatment that otherwise do not have an indication for earlier delivery.

This recommendation factors in the risks of stillbirth, neonatal morbidity, and infant death by calculating the comparative risk of delivery in a given vs subsequent week of gestation. At 39 completed weeks of gestation, the risk of delivery in the subsequent week of gestation statistically increased for pregnancies conceived with infertility treatment (120 per 10 000 live births) and with ART specifically (154 per 10 000 live births). This finding equated to a number needed to deliver of 83 for pregnancies conceived with infertility treatment and 64 for pregnancies conceived with ART to prevent 1 excess neonatal morbidity or infant death. Our recommendation for timing of delivery, at 39 completed weeks of gestation, is consistent with recommendations from the ARRIVE (A Randomized Trial of Induction Versus Expectant Management) trial, an interventional study of term induction vs expectant management.^17^ In the ARRIVE trial of low-risk nulliparous women, delivery at 39 weeks’ gestation resulted in decreased perinatal comorbidities (4.3% vs 5.4%; RR, 0.80 [95% CI, 0.64-1.00]). Regarding the cost-effectiveness of induction of labor, an economic analysis in 5 Utah hospitals showed that costs of elective labor induction did not differ substantially from expectant management.^18^ When additional costs of stillbirth or NICU admission incurred by delivery at a later gestational age are tallied into the balance, elective induction likely will be more economical.

Other studies that analyzed stillbirth in patients using infertility treatment collectively reaffirmed the increased risk of stillbirth and neonatal morbidity.^19,20,21,22^ A meta-analysis that included 10 studies reported an increased risk of stillbirth (odds ratio, 1.82; 95% CI, 1.37-2.42) for pregnancies conceived with IVF and intracytoplasmic sperm injection (ICSI) compared with spontaneous pregnancies.^19^ A study from Denmark from 1989 to 2006 found that the risk of stillbirth in pregnancies conceived with IVF or ICSI was 1.62%, and pregnancies conceived with non-IVF or non-ICSI treatment had a lower risk at 0.23%.^20^ Another US study using the NCHS natality and fetal death files from 2014 to 2017 found a significantly increased hazard ratio of 1.21 for a stillbirth outcome among patients who used any infertility treatment.^21^ Additionally, a 2016 meta-analysis of 50 cohort studies revealed that pregnancies conceived with infertility treatment were at a significantly higher risk for low birth weight, very low birth weight, and perinatal mortality outcomes.^22^ These studies reiterated the importance of increased monitoring and the need for tailored delivery timing in this at-risk patient population. Prior models proposed by Rosenstein et al^9^ in low-risk term pregnancies examined the perinatal outcomes of stillbirth and infant death to optimize the timing of delivery. To fully capture the risk of delivery in the subsequent week of gestation, we accounted for neonatal morbidity, which at term gestation is an important component of perinatal outcomes. Thus, to our knowledge, the present study is the first to examine rates of stillbirth, neonatal morbidity, and infant death in the same time point of each week of term gestation in pregnancies conceived with infertility treatment.^17^

### Strengths and Limitations

A major strength of this study was the use of a large contemporary US data set that allowed for the examination of stillbirth and infant death rates at each week of term gestation, which are low frequency but impactful events. Another strength of the study was the inclusion of pregnancies conceived with infertility treatment and the subgroup analysis focusing on pregnancies conceived with ART. This inclusion allowed for the additional study of potential causes of infant deaths and stillbirths in at-risk pregnancies. The definitions of infertility treatment and ART were the same as those used by the American Society for Reproductive Medicine. Our ability to exclude additional common comorbidities, such as chronic hypertension, preeclampsia, and diabetes, focused the question of timing of delivery on pregnancies conceived with infertility treatment. These conditions are known to be factors in perinatal mortality and neonatal morbidity risk and to have established guidelines regarding delivery timing.

The limitations of the study included its retrospective design and lack of data regarding intent or indication for delivery or whether antenatal fetal surveillance was used in these pregnancies. Another limitation was that all stillbirths were assumed to have occurred during the week in which they were delivered, but the stillbirth may have occurred earlier. Given that the standard obstetrical care in the US includes the practice of weekly prenatal visits at term with fetal heart rate auscultation, this is a judicious assumption, but one that could skew the stillbirth risk toward later gestations. Therefore, restraint must be exercised when making clinical recommendations based on these data, and the findings must be cautiously interpreted and explored further. Despite these limitations, this study was population-based and thus was representative of a contemporary cohort of patients who received infertility treatment in the US.

## Conclusions

In this cohort study, delivery at 39 weeks was associated with the lowest perinatal risk when comparing the risk of delivery at a given week vs subsequent week of gestation. The findings suggest an increased risk of adverse perinatal outcomes with both early-term and late-term delivery among patients who conceived with infertility treatment.

## Notes

Supplement 1. **eTable 1.** Baseline Patient Characteristics in Spontaneous Pregnancies and in Pregnancies Resulting From Infertility Treatment and Specifically ART **eTable 2.** Comparative Risks of Stillbirth, Infant Death, Infant Death or Neonatal Morbidity by Gestational Age in Pregnancies Resulting From Infertility Treatment **eTable 3.** Comparative Risks of Stillbirth, Infant Death by Gestational Age in Pregnancies Resulting From Assisted Reproductive Therapy **eTable 4.** Causes of Infant Death by Gestational Age, 37-42 Weeks, in Pregnancies Resulting From Infertility Treatment **eTable 5.** Initiating Attributable Causes of Stillbirth by Gestational Age, 37-42 Weeks, in Pregnancies Resulting From Infertility Treatment **eFigure 1.** Study Population **eFigure 2.** Thematically Grouped Causes of Infant Death (A) and Stillbirths (B) in Pregnancies Resulting From Any Infertility Treatment

Supplement 2.
Data Sharing Statement
