# Supplementary material for: Gestational Age and Birth Outcomes in Term Singleton Pregnancies Conceived With Infertility Treatment
Source: JAMA Netw Open. 2023 Aug 11;6(8):e2328335. doi: 10.1001/jamanetworkopen.2023.28335 (PMC10422180; doi:10.1001/jamanetworkopen.2023.28335)
Supplement: Supplement 1. — eTable 1. Baseline Patient Characteristics in Spontaneous Pregnancies and in Pregnancies Resulting From Infertility Treatment and Specifically ART eTable 2. Comparative Risks of Stillbirth, Infant Death, Infant Death or Neonatal Morbidity by Gestational Age in Pregnancies Resulting From Infertility Treatment eTable 3. Comparative Risks of Stillbirth, Infant Death by Gestational Age in Pregnancies Resulting From Assisted Reproductive Therapy eTable 4. Causes of Infant Death by Gestational Age, 37-42 Weeks, in Pregnancies Resulting From Infertility Treatment eTable 5. Initiating Attributable Causes of Stillbirth by Gestational Age, 37-42 Weeks, in Pregnancies Resulting From Infertility Treatment eFigure 1. Study Population eFigure 2. Thematically Grouped Causes of Infant Death (A) and Stillbirths (B) in Pregnancies Resulting From Any Infertility Treatment [file jamanetwopen-e2328335-s001.pdf]

Supplemental Online Content

Hamilton I, Martin N, Liu J, DeFranco E, Rossi R. Gestational age and birth outcomes in term singleton pregnancies conceived with infertility treatment. *JAMA Netw Open*. 2023;6(8):e2328335. doi:10.1001/jamanetworkopen.2023.28335

eTable 1. Baseline Patient Characteristics in Spontaneous Pregnancies and in Pregnancies Resulting From Infertility Treatment and Specifically ART

eTable 2. Comparative Risks of Stillbirth, Infant Death, Infant Death or Neonatal Morbidity by Gestational Age in Pregnancies Resulting From Infertility Treatment

eTable 3. Comparative Risks of Stillbirth, Infant Death by Gestational Age in Pregnancies Resulting From Assisted Reproductive Therapy

eTable 4. Causes of Infant Death by Gestational Age, 37-42 Weeks, in Pregnancies Resulting From Infertility Treatment

eTable 5. Initiating Attributable Causes of Stillbirth by Gestational Age, 37-42 Weeks, in Pregnancies Resulting From Infertility Treatment

eFigure 1. Study Population

eFigure 2. Thematically Grouped Causes of Infant Death (A) and Stillbirths (B) in Pregnancies Resulting From Any Infertility Treatment

This supplemental material has been provided by the authors to give readers additional information about their work.

© 2023 Hamilton I et al. *JAMA Network Open.*

eTable 1. Baseline Patient Characteristics in spontaneous pregnancies and in pregnancies resulting from infertility treatment and specifically ART

Characteristics	Pregnancies resulting from spontaneous conception	Pregnancies resulting from infertility treatment	Pregnancies resulting from ART
Ethnicity/race, n (%)			
Hispanic	3,599,959 (24.3)	13,957 (8.0)	7,204 (7.8)
Non-Hispanic Black	1,960,570 (13.2)	7,091 (4.1)	3,799 (4.1)
Non-Hispanic White	7,826,250 (52.8)	131,041 (74.9)	66,593 (72.3)
Other	1,444,679 (9.7)	22,810 (13.0)	14,506 (15.8)
Age, y	28.5 ± 5.7	34.2 ± 5.2	35.6 ± 5.1nulli
Advanced maternal age (≥35Y)	2,368,144 (15.8)	81,783 (45.8)	53,619 (56.5)
Nulliparity	4,675,173 (34.8)	75,610 (53.3)	39,996 (52.42)
Less than high school diploma	2,024,807 (13.7)	2,242 (1.3)	931 (1.0)
Married	8,625,022 (60.9)	157,192 (92.7)	81,540 (92.8)
Women, Infants and Children Enrollment	5,804,199 (39.4)	10,395 (5.9)	3,952 (4.2)
Prenatal care			
No Prenatal Care	202,280 (1.4)	255 (0.2)	149 (0.2)
Late initiation (>20 weeks' gestation)	1,945,649 (13.4)	6,943 (4.0)	3,732 (0.2)
Tobacco use	997,916 (6.7)	1,395 (0.8)	400 (0.4)
BMI (mean)	26.4 ± 6.3	25.9 ± 5.9	25.2 ± 5.4
Obesity (BMI ≥ 30)	3,473,587 (23.9)	35,986 (20.5)	15,476 (16.6)
Gestational age (weeks)	39.2 ± 1.2	39.2 ± 1.2	39.1 ± 1.2
Birthweight (g)	3,384 ± 461	3,415 ± 468	3,425 ± 471
SGA, n (%)	1,405,096 (9.4)	14,784 (8.4)	7,642 (8.1)

p value <0.05; BMI: Body Mass Index; SGA: small for gestational age

Results are displayed in the table as a percent (%) or a mean (+/- standard deviation)

NA: Not applicable, not recorded in the fetal death data

Other includes American Indian or Alaskan, Asian, Pacific Islander

© 2023 Hamilton I et al. *JAMA Network Open.*

eTable 2. Comparative risks of stillbirth, infant death, infant death or neonatal morbidity by gestational age in pregnancies resulting from infertility treatment

Week of gestation	Stillbirth Risk (95% CI)	Infant Death Risk (95% CI)	Risk of infant death or neonatal morbidity (95% CI)	Mortality risk of delivery in subsequent week of gestation (95% CI)	Risk of delivery in subsequent week of gestation*(95% CI)	Comparative mortality risk of delivery in subsequent week of gestation compared to delivery (95% CI)	Comparative risk of mortality and neonatal morbidity with delivery in subsequent week of gestation compared to delivery (95% CI)
37	Reference	1.56 (0.90-1.69)	2.10 (1.98-2.22)	Reference	Reference	0.90 (0.50-1.63)	0.62 (0.59-0.66)
38	1.4 (1.0-1.9)	0.94 (0.55-1.61)	1.30 (1.23-1.38)	1.16 (0.75-1.79)	0.77 (0.73-0.81)	1.72 (1.04-2.86)	0.77 (0.73-0.82)
39	1.5 (1.1-2.2)	Reference	Reference	1.16 (0.73-1.86)	0.95 (0.90-1.01)	1.63 (1.08-2.47)	1.25 (1.19-1.31)
40	1.9 (1.3-2.9)	0.93 (0.57-1.51)	1.24 (1.18-1.31)	1.66 0.99-2.79)	1.02 (0.95-1.09)	2.51 (1.48-4.26)	1.08 (1.00-1.15)
41	2.6 (1.5-4.4)	1.46 (0.84-2.52)	1.32 (1.24-1.41)	1.59 (0.67-3.75)	1.12 (0.99-1.26)	1.53 (0.62-3.78)	1.11 (0.98-1.25)
42	6.8 (3.6-13.8)	1.05 (0.33-3.38)	1.45 (1.29-1.62)	-	-		

* Risk of delivery in subsequent week of gestation = risk of stillbirth at this gestational age + risk of infant death or serious neonatal morbidity at the next gestational age week
Data are relative risk (95% confidence interval)

© 2023 Hamilton I et al. *JAMA Network Open.*

eTable 3. Comparative risks of stillbirth, infant death by gestational age in pregnancies resulting from assisted reproductive therapy

Week of gestation	Stillbirth Risk (95% CI)	Infant Death Risk (95% CI)	Risk of infant death or neonatal morbidity (95% CI)	Mortality risk of delivery in subsequent week of gestation (95% CI)	Risk of delivery in subsequent week of gestation*(95% CI)	Comparative mortality risk of delivery in subsequent week of gestation compared to delivery (95% CI)	Comparative risk of mortality and neonatal morbidity with delivery in subsequent week of gestation compared to delivery (95% CI)
37	Reference	2.40 (1.16-4.99)	0.75 (0.39-1.45)	Reference	Reference	0.70 (0.33-1.52)	0.63 (0.58-0.68)
38	1.9 (1.1-3.1)	1.20 (0.56-2.61)	0.85 (0.51-1.42)	1.13 (0.61-2.10)	0.77 (0.72-0.83)	1.58 (0.78-3.20)	0.77 (0.72-0.83)
39	2.1 (1.2-3.5)	Reference	Reference	1.33 (0.69-2.54)	0.99 (0.92-1.07)	2.24 (1.23-4.10)	1.30 (1.22-1.39)
40	2.1 (1.1-3.8)	1.24 (0.62-2.49)	1.39 (0.75-2.59)	1.84 (0.90-3.77)	2.14 (1.99-2.32)	2.52 (1.24-5.15)	1.01 (0.92-1.10)
41	3.1 (1.5-6.6)	2.11 (1.00-4.47)	1.36 (0.48-3.89)	1.80 (0.60-5.46)	1.04 (0.89-1.21)	1.45 (0.46-4.53)	1.05 (0.90-1.24)
42	9.3 (3.8-22.8)	1.52 (0.35-6.54)	-	-	-	-	-

*Risk of delivery in subsequent week of gestation = risk of stillbirth at this gestational age + risk of infant death or serious neonatal morbidity at the next gestational age week
Data are relative risk (95% confidence interval)

© 2023 Hamilton I et al. *JAMA Network Open.*

eTable 4. Causes of infant death by gestational age, 37-42 weeks, in pregnancies resulting from infertility treatment

Gestational Age (week)							
	37	38	39	40	41	42	Total
Infant Death Category							
SIDS	2 (11.1)	4 (21.0)	3 (6.8)	0	2 (11.1)	0	11 (8.6)
Accidents/Trauma	1 (5.6)	2 (10.5)	1 (2.3)	1 (3.8)	2 (11.1)	0	7 (5.5)
Related to labor/delivery	1 (5.6)	3 (15.8)	6 (13.6)	3 (11.5)	2 (11.1)	0	15 (11.7)
Infection	1 (5.6)	0	4 (9.1)	4 (15.4)	0	1 (25.0)	9 (7.0)
Pulmonary	1 (5.6)	0	3 (6.8)	1 (3.8)	0	0	5 (3.9)
Cardiac	3 (16.7)	0	4 (9.1)	3 (11.5)	3 (16.7)	0	13 (10.2)
Neoplasm	1 (5.6)	0	1 (2.3)	0	0	0	2 (1.6)
Other	8 (44.4)	9 (47.4)	20 (45.5)	11 (42.3)	8 (44.4)	3 (75.0)	59 (46.1)
Total	18	19	44	26	18	4	128

Data are n (%) or n

© 2023 Hamilton I et al. *JAMA Network Open.*

eTable 5. Initiating attributable causes of stillbirth by gestational age, 37-42 weeks, in pregnancies resulting from infertility treatment

Gestational Age (week)							
	37	38	39	40	41	42	Total
Stillbirth Category							
Placental abruption	2 (3.6)	4 (6.0)	4 (6.6)	3 (7.9)	0	0	13 (10.2)
Placental abnormalities	2 (3.6)	5 (7.4)	4 (6.6)	0	1 (5.6)	0	12 (9.4)
Cord accident	11 (20.0)	18 (26.9)	18 (29.5)	11 (28.9)	2 (11.1)	2 (22.2)	62 (25.0)
Labor	2 (3.6)	3 (4.5)	1 (1.6)	1 (2.6)	1 (5.6)	0	8 (3.2)
Infection	1 (1.8)	2 (3.0)	0	2 (5.3)	1 (5.6)	0	6 (2.4)
Maternal medical complications	5 (9.1)	1 (1.5)	1 (1.6)	0	0	2 (22.2)	9 (3.6)
Unknown	41.8)	26 (38.8)	30 (49.2)	17 (44.7)	9 (50.0)	5 (55.6)	110 (44.4)
Other	9 (16.4)	8 (11.9)	3 (4.9)	4 (10.5)	4 (22.2)	0	28 (11.3)
Total	55	67	61	38	18	9	248

Data are n(%) or n

© 2023 Hamilton I et al. *JAMA Network Open.*

eFigure 1. Study Population

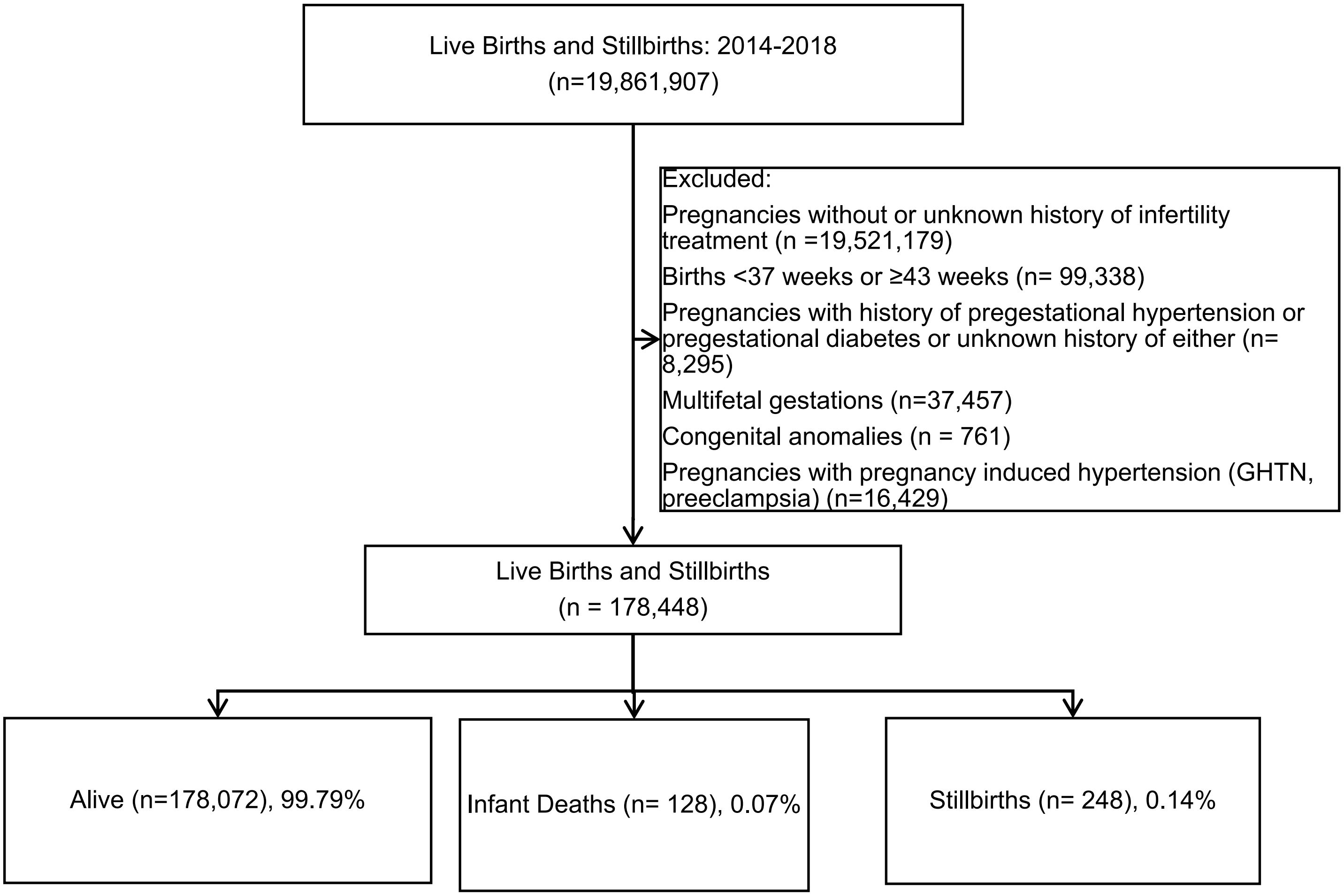

© 2023 Hamilton I et al. *JAMA Network Open.*

eFigure 2. Thematically grouped causes of infant deaths (A) and stillbirths (B) in pregnancies resulting from any infertility treatment

A.

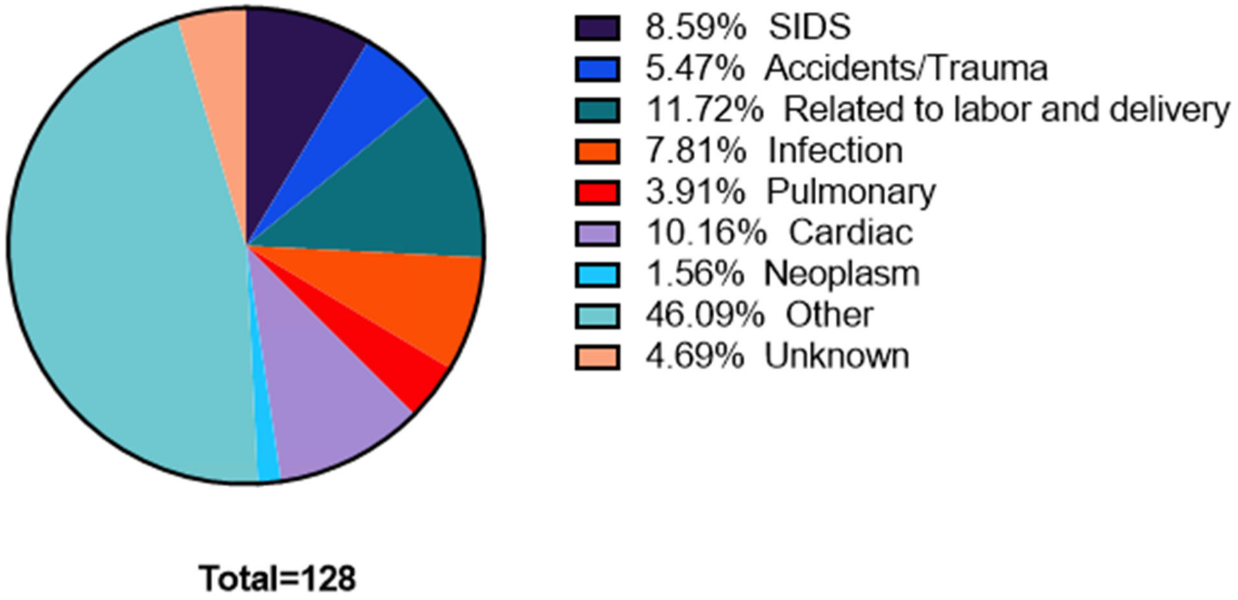

B.

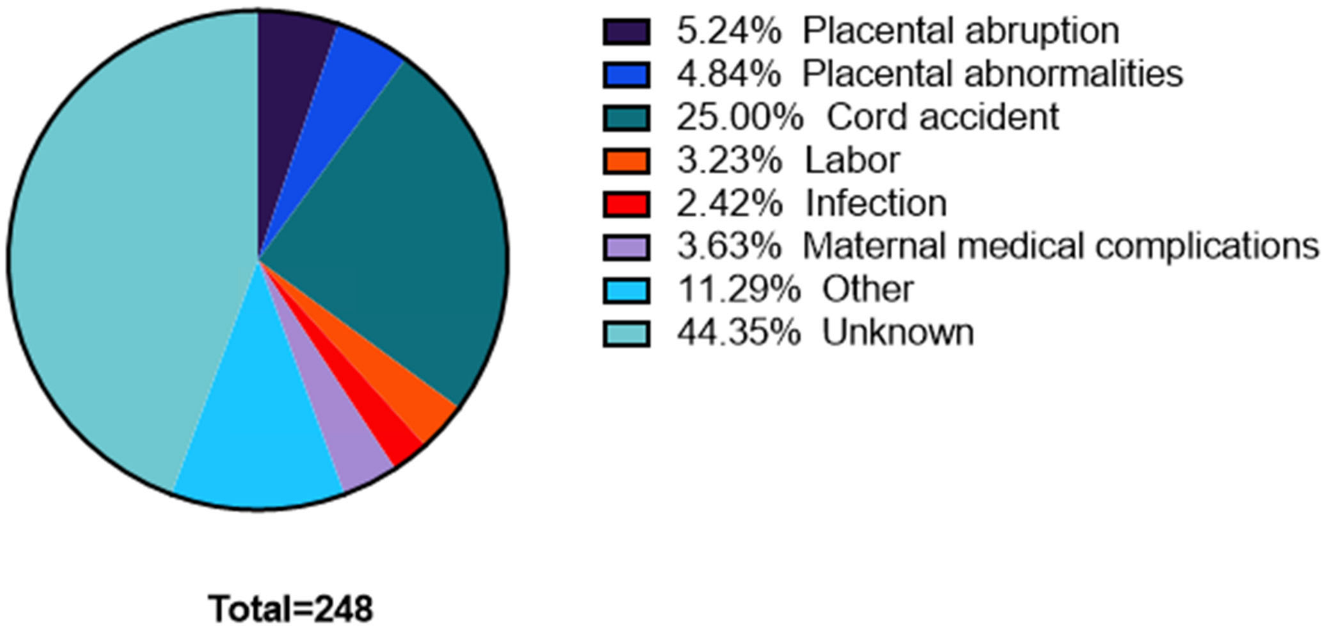

© 2023 Hamilton I et al. *JAMA Network Open.*
