# Supplementary material for: Gestational Age and Birth Outcomes in Term Singleton Pregnancies Conceived With Infertility Treatment
Source: JAMA Netw Open. 2023 Aug 11;6(8):e2328335. doi: 10.1001/jamanetworkopen.2023.28335 (PMC10422180; doi:10.1001/jamanetworkopen.2023.28335)
Supplement: Supplement 2. — Data Sharing Statement [file jamanetwopen-e2328335-s002.pdf]

Data Sharing Statement

Hamilton. Gestational Age and Birth Outcomes in Term Singleton Pregnancies Conceived With Infertility Treatment. *JAMA Netw Open*. Published August 11, 2023. doi:10.1001/jamanetworkopen.2023.28335

Data
Data available: No
